# A Novel Strategy for High Quantum Efficiency Composite Oxide Far-Red Phosphors: Ca_14_Mg_5.94_Li_0.03_In_0.03_Ga_9.95_O_35_:0.05Mn^4+^

**DOI:** 10.3390/ma19071367

**Published:** 2026-03-30

**Authors:** Juan Li, Huiying Ye, Fachangsheng Zhong, Peng Wu, Menghao Chang, Linkun Han, Jingwu Zheng, Liang Qiao, Jing Yu, Yao Ying, Wei Cai, Shenglei Che

**Affiliations:** 1 College of Materials Science and Engineering, Zhejiang University of Technology, Hangzhou 310014, China; 211123250046@zjut.edu.cn (H.Y.); 221124250112@zjut.edu.cn (F.Z.); 211124250037@zjut.edu.cn (P.W.); 221124250208@zjut.edu.cn (M.C.); 221124250221@zjut.edu.cn (L.H.); zhengjw@zjut.edu.cn (J.Z.); lqiao@zjut.edu.cn (L.Q.); yujing@zjut.edu.cn (J.Y.); yying@zjut.edu.cn (Y.Y.); caiwei@zjut.edu.cn (W.C.); cheshenglei@zjut.edu.cn (S.C.); 2 Research Center of Magnetic and Electronic Materials, Zhejiang University of Technology, Hangzhou 310014, China

**Keywords:** phosphor, far-red emission, plant growth, Mn^4+^, synthesizing

## Abstract

Far-red phosphors featuring high quantum efficiency and emission bands that strongly overlap with the absorption spectra of plant pigments are crucial for advancing plant cultivation lighting technology. Restricted by the large Stokes shift, far-red phosphors typically exhibit low energy efficiency. Moreover, many far-red phosphors suffer from low quantum efficiency, which has emerged as a critical issue in the research of these materials. To address the issue, conventional strategies—including crystal field engineering, defect engineering, and sensitizer doping—have been widely adopted to enhance their emission intensity. In this work, we propose a novel and effective strategy to improve the emission performance of far-red phosphors: low-melting-point magnesium chloride has been introduced as a flux to regulate the reaction pathway of the composite oxide phosphor Ca_14_Mg_5.94_Li_0.03_In_0.03_Ga_9.95_O_35_:0.05Mn^4+^ (CMLIGO:0.05Mn^4+^). The cubic intermediate product with a structure analogous to the target product has been designed to form a compact lattice structure and reduce crystal defects, thereby enhancing the luminescence intensity and quantum efficiency of the phosphor. The Ca_14_Mg_5.94_Li_0.03_In_0.03_Ga_9.95_O_35_:0.05Mn^4+^@3 wt% MgCl_2_ (CMLIGO:0.05Mn^4+^@3 wt% MgCl_2_) shows a broad excitation band (250–600 nm) and far-red emission centered at 720 nm (650–800 nm). Under 365 nm excitation, the CMLIGO:0.05Mn^4+^@3 wt% MgCl_2_ exhibits an internal quantum efficiency of 91.4%. Benefiting from its high internal quantum efficiency and the emission band that matches well with the absorption spectrum of phytochrome in the far-red absorbing form (phytochrome P_fr_), CMLIGO:0.05Mn^4+^@3 wt% MgCl_2_ demonstrates promising potential for applications in plant cultivation lighting. This work offers a new direction for synthesizing and modification of composite oxide phosphors.

## 1. Introduction

Plant cultivation lighting is a key technology in indoor farming [1]. Providing highly customized and reliable light sources for plants helps improve both the yield and quality of agricultural products [2,3]. Phosphor-converted light-emitting diodes (pc-LEDs), known for their low energy consumption and highly tunable emission color, have been widely applied for indoor cultivation. Compared to traditional fluorescent or high-pressure sodium (HPS) lamps, pc-LEDs generate less heat and can significantly mitigate heat stress in plants [4,5,6].

The essential light spectra for plant growth correspond to the absorption ranges of specific photoreceptors: blue light (400–500 nm), absorbed by chlorophyll A and B; red light (600–700 nm), absorbed by the red-light-absorbing form of phytochrome (phytochrome P_r_); and far-red light (700–750 nm), absorbed by the far-red-light-absorbing form of phytochrome (phytochrome P_fr_). To match the absorption spectra of plant pigments, pc-LEDs for plant cultivation lighting are typically fabricated by combining blue LED chips with red phosphors and far-red phosphor [7]. Currently, red phosphors with high quantum efficiency and an emission peak in the range of 600–630 nm are commercially available, such as MAlSiN_3_:Eu^2+^ (M = Ca, Sr) [8,9] and M_2_Si_5_N_8_:Eu^2+^ (M = Ca, Sr, Ba) [10,11]. However, research on far-red phosphors remains relatively limited. Therefore, it is of great significance to develop far-red phosphors that exhibit both high quantum efficiency and wide spectral overlap with the absorption band of phytochrome P_fr_.

Cr^3+^ and Mn^4+^ are the predominant activator ions investigated for far-red phosphors. Compared with the activator ions that emit in the red region (e.g., Eu^3+^ and Sm^3+^) or in the near-infrared region (e.g., Pr^3+^), both Cr^3+^ and Mn^4+^ are more cost-effective and exhibit emission spectra that align well with the absorption band of phytochrome P_fr_. Cr^3+^ possesses a 3d^3^ electronic configuration. Its emission is strongly influenced by the crystal field. By selecting appropriate hosts, both narrow-band [12,13] and broad-band [14,15] emissions of Cr^3+^ in the far-red region can be achieved. Mn^4+^ also features a 3d^3^ electronic configuration. However, compared to Cr^3+^, the emission band of Mn^4+^ exhibits higher predictability, typically characterized by a combination of the zero-phonon line (ZPL) and phonon sidebands. When introduced as activator ions for far-red emission, both Cr^3+^ and Mn^4+^ commonly occupy octahedral sites in the host lattice.

Based on these, the hosts for far-red phosphors mainly include those with perovskite or double perovskite structures [16,17,18], garnet structure [19,20], β-Ca_3_(PO_4_)_2_ structure [21,22], spinel structure [23], or β-Al_2_O_3_ structure [24]. These structures share a common feature: they possess abundant octahedral sites available for doping with Mn^4+^ or Cr^3+^. Furthermore, the double perovskite structure also contains dodecahedral and tetrahedral sites, which can accommodate sensitizer ions or ions for crystal field engineering. Research on double perovskite phosphors has led to the development of numerous far-red phosphors based on niobate [25], tungstate [26] or tantalate [27] hosts. Garnets, with the longest history as phosphor hosts, similarly feature dodecahedral and tetrahedral sites for the doping of various elements [28,29]. Hosts with a garnet structure exhibit excellent structural stability. Compounds with the β-Ca_3_(PO_4_)_2_ [30] structure serve as excellent host materials for phosphors due to their thermal stability and the availability of six distinct cationic sites for ion substitution. The spinel structure, which comprises both octahedral and tetrahedral sites, offers a less diverse array of available sites. However, its cubic symmetry is advantageous for the development of fluorescent ceramics [31]. The β-Al_2_O_3_ structure can be described as consisting of spinel blocks separated by conduction planes. While this structure accommodates the substitution of various ions, its inherently loose framework generally makes it a suboptimal choice as a phosphor host.

Tululite [32] represents a family of cubic oxides with the general chemical formula Ca_14_(A, B)_15_O_35+x_ (0 ≤ x ≤ 1) (A = Fe, Al; B = Al, Zn, Fe, Si, P, Mn, Mg). Benefiting from its flexible composition, the crystal field of tululite is highly sensitive to minor variations in chemical composition, thereby providing a complex and tunable crystal field environment for activator ions. Additionally, the tululite structure features a diversity of interstitial sites, including decahedral, octahedral, and tetrahedral coordinations, which can accommodate various ions such as Mn^4+^, Cr^3+^, and other ions for lattice modification. These advantages make tululite a promising host material for phosphors.

To date, most research on tululite-based phosphors has focused on Ca_14_Zn_6_Ga_10_O_35_ and Ca_14_Zn_6_Al_10_O_35_, which can be activated by Mn^4+^ [33,34] or Cr^3+^ [35,36] to produce far-red emission. Both Ca_14_Zn_6_Ga_10_O_35_ and Ca_14_Zn_6_Al_10_O_35_ exhibit a strong crystal field, which leads to narrow far-red emission in their Cr^3+^-doped samples. This sharp emission shows limited spectral overlap with the absorption band of phytochrome P_fr_. In contrast, Mn^4+^ serves as a more suitable far-red activator for tululite-type hosts.

Ca_14_Mg_6_Ga_10_O_35_ emerges as a novel member of the tululites. When doped with Mn^4+^, Ca_14_Mg_6_Ga_10_O_35_ can be excited by UV light at 300 nm and exhibits a broad emission band from 650 to 800 nm, peaking at 720 nm. In contrast, the emission peaks of Ca_14_Zn_6_Al_10_O_35_:Mn^4+^ [37,38] and Ca_14_Zn_6_Ga_10_O_35_:Mn^4+^ [39,40] are located at 710 nm and 712 nm, respectively. The emission profile of Ca_14_Mg_6_Ga_10_O_35_:Mn^4+^ shows a superior overlap with the absorption spectrum of phytochrome P_fr_, presenting its potential as a phosphor for plant cultivation lighting.

The performance of far-red pc-LEDs heavily relies on the luminescence intensity of the phosphors used. To date, three strategies have been employed to enhance phosphor luminescence: (1) crystal field engineering [41,42,43], modifying the local crystal field around activator ions through ionic substitution to reduce the probability of non-radiative transitions; (2) introducing sensitizer ions and establishing efficient energy transfer from sensitizers to activators [44,45,46]; (3) defect engineering [47,48,49], controlling the density of lattice defects, particularly those that act as quenching centers, to minimize non-radiative decay. The common defect engineering methods include regulating the calcination atmosphere (e.g., using a reducing environment) or doping with ions of specific charge states [50].

However, the intermediate products formed in the Ca_14_Mg_6_Ga_10_O_35_ system when synthesizing samples by a high-temperature solid-state reaction are Ca_5_Ga_6_O_14_ and Ca_3_Ga_4_O_9_. Both of them possess a layered structure, whose integration into the CMGO tends to introduce defects that can act as quenching centers [51]. In our previous work on Ca_14_Zn_6_Ga_10_O_35_ [52], it was discovered that certain additives can change the types of intermediate products during the reaction. Based on it, this work designs a reaction pathway with new intermediate products which have crystal structures more similar to the CMGO with a cubic structure. The adjusted approach will reduce lattice defects in the final samples and improve the emission intensity. This work offers a new strategy for the synthesis and modification of complex oxide phosphors.

In our earlier unpublished work, we obtained Ca_14_Mg_6_Ga_9.95_O_35_:0.05Mn^4+^ (CMGO:0.05Mn^4+^) exhibiting far-red emission by doping Mn^4+^ into Ca_14_Mg_6_Ga_10_O_35_. Subsequently, we attempted to further enhance the luminescence intensity through crystal field engineering. By substituting Li^+^-In^3+^ ion pairs for Mg^2+^-Mg^2+^ ion pairs, we obtained Ca_14_Mg_5.94_Li_0.03_In_0.03_Ga_9.95_O_35_:0.05Mn^4+^ (CMLIGO:0.05Mn^4+^) with improved photoluminescence intensity.

In this work, the low-melting-point chloride salt MgCl_2_ was introduced to alter the reaction pathway of Ca_14_Mg_5.94_Li_0.03_In_0.03_Ga_9.95_O_35_:0.05Mn^4+^ by promoting the formation of cubic intermediate products during synthesis and thereby improve its emission intensity.

## 2. Experimental

### 2.1. Synthesis

The phosphors Ca_14_Mg_6_Ga_9.95_O_35_:0.05Mn^4+^ (CMGO:0.05Mn^4+^) and Ca_14_Mg_5.94_Li_0.03_In_0.03_Ga_9.95_O_35_:0.05Mn^4+^@*x* wt% MgCl_2_(CMLIGO:0.05Mn^4+^@*x* wt% MgCl_2_) were synthesized via traditional high-temperature solid-state reaction with CaCO_3_ (99.99%, Macklin, Shanghai, China), 4MgCO_3_·Mg(OH)_2_·5H_2_O (99.7%, Shanghai Silian Chemical Factory Co., Ltd., Shanghai, China), Ga_2_O_3_ (99.99%, Aladdin, Shanghai, China), MnCO_3_ (99.99%, Aladdin, Shanghai, China), Li_2_CO_3_ (99.99%, Aladdin, Shanghai, China) and In_2_O_3_ (99.99%, Aladdin, Shanghai, China). MgCl_2_ (99.9%, Rhawn, Shanghai, China) was introduced into the initial mixtures of raw materials as a flux. The additive amount of MgCl_2_ was relative to the total mass of the mixed raw materials which is 0 wt%, 2 wt%, 3 wt%, 4 wt%, 5 wt%, 6 wt%, respectively.

All of the raw materials were weighed according to the stoichiometric quantities and mixed with or without the flux for 60 min. The mixtures were placed in an alumina boat and calcined in a tube furnace under an air atmosphere. The samples were heated to 1000 °C at a rate of 5 °C/min and held at this temperature for 6 h. After the holding period, samples were heated to 800 °C at a rate of 5 °C/min, followed by natural cooling to room temperature. The resulting powders were ground in an agate mortar for 2.5 min, then transferred back to an alumina boat for a second calcination in the tube furnace under air. The samples were heated to 1275 °C at a rate of 5 °C/min and held for 6 h, then cooled to 800 °C at 5 °C/min, and finally allowed to cool naturally to room temperature. The calcined samples were manually ground again for 2.5 min in an agate mortar to obtain the final Ca_14_Mg_6_Ga_9.95_O_35_:0.05Mn^4+^ and Ca_14_Mg_5.94_Li_0.03_In_0.03_Ga_9.95_O_35_:0.05Mn^4+^@*x* wt% MgCl_2_ powdered samples.

### 2.2. Characterization

All samples were characterized by X-ray diffraction (XRD) using the Rigaku SmartLab SE diffractometer (Rigaku Corporation, Tokyo, Japan) in the range of 10° to 80° with a scanning step size of 0.02°. The crystal structure of Ca_14_Mg_6_Ga_10_O_35_ was visualized with the VESTA software (Version 3.5.8), co-developed by Koichi Momma (National Museum of Nature and Science, Tokyo, Japan) and Fujio Izumi (Kyoto University, Kyoto, Japan). The standard PDF card was also simulated by VESTA. Photoluminescence (PL) and photoluminescence excitation (PLE) spectra were acquired using a Hitachi F-4600 fluorescence spectrophotometer (Hitachi High-Tech Science Corporation, Tokyo, Japan). The morphology of the samples was examined by field-emission scanning electron microscopy (NOVA NANOSEM 450, FEI Company, Hillsboro, OR, USA). Simultaneous thermogravimetric and differential scanning calorimetry (TG-DSC) analyses were conducted on a NETZSCH STA 449 F3 analyzer (NETZSCH-Gerätebau GmbH, Selb, Germany). The measurements were carried out in air atmosphere, with a heating rate of 5 °C/min over a temperature range from 30 °C to 1300 °C. The internal quantum yield (IQY) of the phosphors was measured with an Edinburgh Instruments FLS1000 photoluminescence spectrometer (Edinburgh Instruments, Livingston, UK), equipped with an integrating sphere. Thermoluminescence (TL) spectra were measured using an LTTL-3DS multifunctional defect fluorescence spectrometer (Rongfan Technology Co., Ltd., Guangzhou, China) with an excitation time of 120 s and a heating rate of 5 °C/s. Temperature-dependent photoluminescence (TDPL) spectra were acquired on an Edinburgh Instruments FLS980 fluorescence spectrometer (Edinburgh Instruments, Livingston, UK). The TDPL spectra were performed under 365 nm excitation and monitored over the temperature range of 25–200 °C.

## 3. Results and Discussion

### 3.1. Crystal Structure

**Figure 1:**
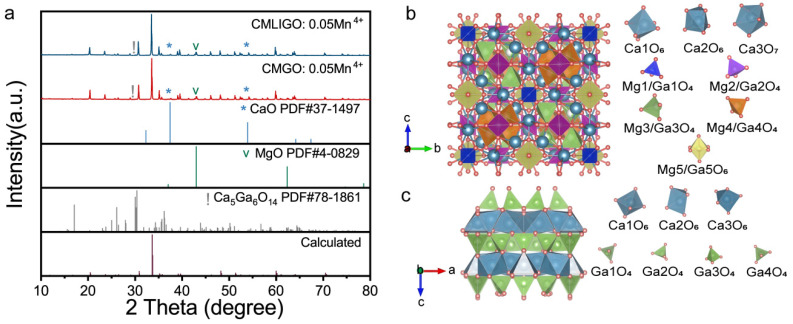
Structure and XRD patterns of CMGO:0.05Mn^4+^ and CMLIGO:0.05Mn^4+^. (**a**) XRD patterns of CMGO:0.05Mn^4+^ and CMLIGO:0.05Mn^4+^. The calculated powder diffraction data was simulated using the unit cell of Ca_14_Mg_6_Ga_10_O_35_ in VESTA. (**b**) Crystal structure of the Ca_14_Mg_6_Ga_10_O_35_ host and the coordination configurations of Ca, Mg, and Ga atoms with oxygen. (**c**) Crystal structure of Ca_5_Ga_6_O_14_ and the coordination configurations of Ca and Ga atoms with oxygen.

Figure 1a presents the XRD patterns of CMGO:0.05Mn^4+^ and CMLIGO:0.05Mn^4+^. The reference powder diffraction data was simulated using the VESTA software package. The simulation employed the structural model of Ca_14_Mg_6_Ga_10_O_35_ in which the atomic coordinates were adopted from Khoury et al.’s report on tululite [32]. In this cubic model, the cation sites are occupied by Ca, Mg, and Ga and the anion sites by O. The lattice constants of the model were refined based on the experimental XRD data.

The crystal structure of Ca_14_Mg_6_Ga_10_O_35_ is depicted in Figure 1b. Ca_14_Mg_6_Ga_10_O_35_ crystallizes in the F23 space group with eight distinct interstitial sites. Here, two types of Ca coordinate with oxygens to form octahedra [Ca1O_6_] and [Ca2O_6_], respectively, while another Ca forms a decahedron [Ca3O_7_] with oxygens. Meanwhile, the smaller cations, Mg and Ga, occupy both tetrahedral and octahedral interstitial sites surrounded by oxygens in a certain ratio. All these polyhedra collectively constitute the cubic unit cell of Ca_14_Mg_6_Ga_10_O_35_.

As observed, the experimental powder diffraction patterns of CMGO:0.05Mn^4+^ and CMLIGO:0.05Mn^4+^ samples are in good agreement with the calculated powder diffraction data, with only a minimal amount of secondary phase present. The diffraction peaks of these secondary phases correspond to the PDF cards of Ca_5_Ga_6_O_14_, CaO and MgO, respectively. The CaO and MgO are derivatives of the raw materials. Ca_5_Ga_6_O_14_ [53,54] is one of the intermediate products formed during the conversion of the raw materials to Ca_14_Mg_6_Ga_10_O_35_. Different from Ca_14_Mg_6_Ga_10_O_35_, Ca_5_Ga_6_O_14_ exhibits a layered structure composed of alternating two-dimensional network layers of gallium–oxygen (GaO) groups and calcium–oxygen (CaO) groups [55], as illustrated in Figure 1c.

### 3.2. Modification

**Figure 2:**
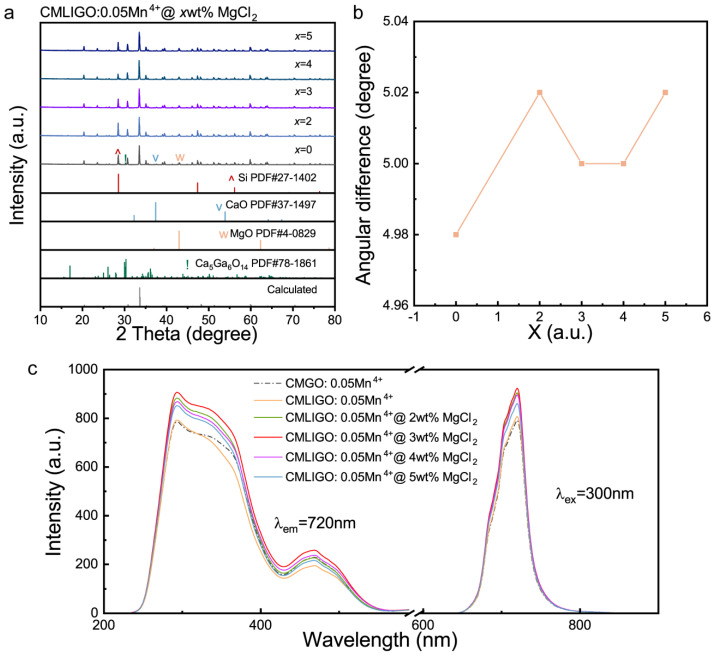
Influence of MgCl_2_ addition on the lattice structure, the excitation and emission spectra. (**a**) XRD patterns of CMLIGO:0.05Mn^4+^@*x* wt% MgCl_2_ (*x* = 0, 2, 3, 4, 5) after the final calcination mixing with Si powder. The high-purity (5N) Si powder was added at a concentration of 12.5 wt% relative to the phosphor samples and served as a standard. For clarity, the XRD curves for the MgCl_2_-added groups are not marked with symbols indicating the three secondary phases and Si, although all samples indeed contained these four phases. (**b**) Variation curves of the angular difference between the strongest diffraction peak of CMLIGO:0.05Mn^4+^@*x* wt% MgCl_2_ and that of high-purity Si powder with *x* (*x* = 0, 2, 3, 4, 5). (**c**) Photoluminescence and photoluminescence excitation spectra of CMGO:0.05Mn^4+^ and CMLIGO:0.05Mn^4+^@*x* wt% MgCl_2_ (*x* = 0, 2, 3, 4, 5). The data for CMGO:0.05Mn^4+^ are included for comparison and are represented by a dash-dotted line.

To enhance the luminescence intensity, MgCl_2_ was introduced to modify CMLIGO:0.05Mn^4+^. The XRD patterns of the samples are shown in Figure 2a. To investigate the shift of CMLIGO:0.05Mn^4+^@*x* wt% MgCl_2_, high-purity (5N, 200 mesh) Si powder was used for calibration, with a constant addition of 12.5 wt% to each sample. The angular difference between the strongest diffraction peak of CMLIGO:0.05Mn^4+^@*x* wt% MgCl_2_ and that of the Si standard is presented in Figure 2b.

In Figure 2a, all samples exhibit only the diffraction peaks corresponding to the target phase, CaO, MgO, Ca_5_Ga_6_O_14_ and the Si standard, with no peaks attributable to MgCl_2_ being detected. The absence of MgCl_2_ peaks could be attributed to its low concentration being below the detection limit or, alternatively, to its incorporation into the crystal lattice. However, as shown in Figure 2b, the XRD peak positions of the samples did not shift systematically with the increasing amount of MgCl_2_, indicating that MgCl_2_ was not incorporated into the crystal lattice. Compared with the pristine CMLIGO:0.05Mn^4+^, the main XRD peak of the MgCl_2_-modified sample exhibited a red shift of 0.02–0.04° in peak position, indicating that the addition of MgCl_2_ induced a lattice contraction.

The PL and PLE spectra of all samples are shown in Figure 2c. The dash-dotted line represents the photoluminescence data of CMGO:0.05Mn^4+^, while the solid line corresponds to that of CMLIGO:0.05Mn^4+^@*x* wt% MgCl_2_ (*x* = 0, 2, 3, 4, 5). All samples exhibit a broad excitation band spanning 200–600 nm and an emission band in the far-red region. It can be observed that the addition of magnesium chloride did not significantly alter the shape of either the PL or PLE spectra of the samples, although it did have an effect on the PL intensity. The peak emission intensity of CMLIGO:0.05Mn^4+^ shows a slight increase compared to that of CMGO:0.05Mn^4+^, and the peak emission intensity of the modified CMLIGO:0.05Mn^4+^@3 wt% MgCl_2_ reaches 115.2% of the value obtained for CMLIGO:0.05Mn^4+^.

**Figure 3:**
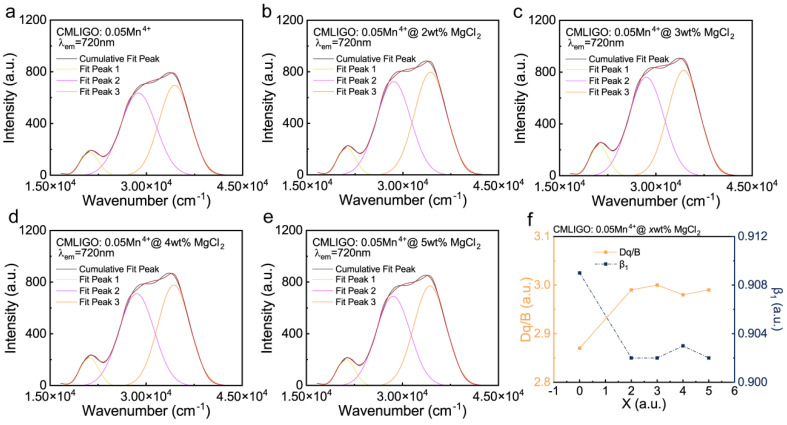
Effect of MgCl_2_ addition on the crystal field. (**a**–**e**) Gaussian deconvolution of the PLE spectra for CMLIGO:0.05Mn^4+^@*x* wt% MgCl_2_ (*x* = 0, 2, 3, 4, 5). (**f**) Variation curves of Dq/B and β_1_ with *x* (*x* = 0, 2, 3, 4, 5) for CMLIGO:0.05Mn^4+^@*x* wt% MgCl_2_.

The PLE spectra can be deconvoluted into four fit peaks via Gaussian fitting on the energy scale as shown in Figure 3a–e: fit peak 1 to the ^4^A_2g_ → ^4^T_2g_ (^4^F) transition; fit peak 2 to the ^4^A_2g_ → ^4^T_1g_ (^4^F) transition. On the high-energy side of fit peak 2, further deconvolution fitting by software proved to be difficult. As previously reported [56], only a single relatively intense peak (fit peak 3) is observed, which can be regarded as a superposition of the CTB absorption and finer absorptions such as the ^4^A_2g_ → ^4^T_1g_ (^4^P) transition. The crystal field parameters for CMLIGO:0.05Mn^4+^@*x* wt% MgCl_2_ were calculated using the following equation [57]:

(1) Dq=EA42g → T42g10

(2) $$\frac{\mathrm{Dq}}{B}=\frac{15\left(x-8\right)}{\left(x^{2}-10x\right)}$$

(3) x=EA2g4 → T1g4−EA2g4 → T2g4Dq

(4) EE2g2 → A2g4B=3.05C/B+7.9−1.8B/Dq

Here, x represents a parameter, while B and C are Racah parameters. The energy values for the ^4^A_2g_ → ^4^T_2g_ (^4^F), ^4^A_2g_ → ^4^T_1g_ (^4^F), and ^2^E_2g_ → ^4^A_2g_ transitions were determined from the peak wavenumbers of the fit peaks for PLE spectra and peak wavenumbers of PL spectra. Dq is the local crystal field strength, related to Racah parameters (B, C) and the energy of the ^2^E_2g_ → ^4^A_2g_ transition by Equation (4).

Furthermore, the parameter β_1_ used to quantitatively describe the covalent effect in hosts was calculated using the following formula [58]:

(5) $$\beta_{1}=\sqrt{{\left(B/B_{0}\right)}^{2}-{\left(C/C_{0}\right)}^{2}}$$

Here, B_0_ and C_0_ are the Racah parameters for the free Mn^4+^ ion, with values of 1160 cm^−1^ and 4303 cm^−1^, respectively. Based on these calculations, the relevant parameters for CMLIGO:0.05Mn^4+^@*x* wt% MgCl_2_ are summarized in Table 1. All parameter β_1_ values are less than 1, exhibiting the characteristic of oxides [59]. The variation of Dq/B and β_1_ as a function of *x* is shown in Figure 3f. As can be seen from Figure 3f, the crystal field strength of all modified samples is enhanced compared to that of the unmodified CMLIGO:0.05Mn^4+^, which corresponds to the shift in the XRD peak positions. That is, the modified sample with MgCl_2_ addition exhibits a more compact lattice and a stronger crystal field. The value of parameter β_1_ is associated with the overlap of wavefunctions between Mn^4+^ and the ligands of the host lattice [57,59]. As shown in Figure 3f, each of the modified samples with MgCl_2_ addition presents a lower β_1_ value, a trend that is consistent with the variation in crystalline interplanar spacing observed in the XRD results.

**Figure 4:**
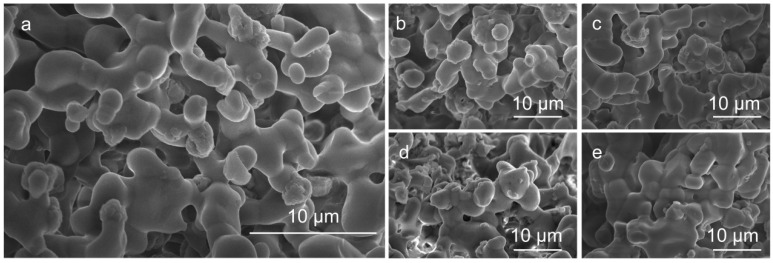
(**a**–**e**) SEM images (magnified 5000 times) of CMLIGO:0.05Mn^4+^@*x* wt% MgCl_2_ with *x* = 0, 2, 3, 4, 5.

Figure 4a–e present SEM images (magnified 5000 times) of CMLIGO:0.05Mn^4+^ samples with varying MgCl_2_ additions. The images reveal that samples with MgCl_2_ contents ranging from 0 to 4 wt% maintain relatively distinct grain boundaries. In contrast, when the MgCl_2_ content exceeds 4 wt%, the grain boundaries become blurred and exhibit a molten morphology. This observation indicates that MgCl_2_ can function as a fluxing agent in the CMLIGO:0.05Mn^4+^ system. Figure S1 presents the SEM images (magnified 1000 times) of samples with varying MgCl_2_ additions, along with the corresponding particle size distribution histograms. It can be observed that, as the amount of MgCl_2_ increases, the proportion of small-sized grains decreases, which confirms the fluxing effect of MgCl_2_.

### 3.3. Analysis of Reaction Path

**Figure 5:**
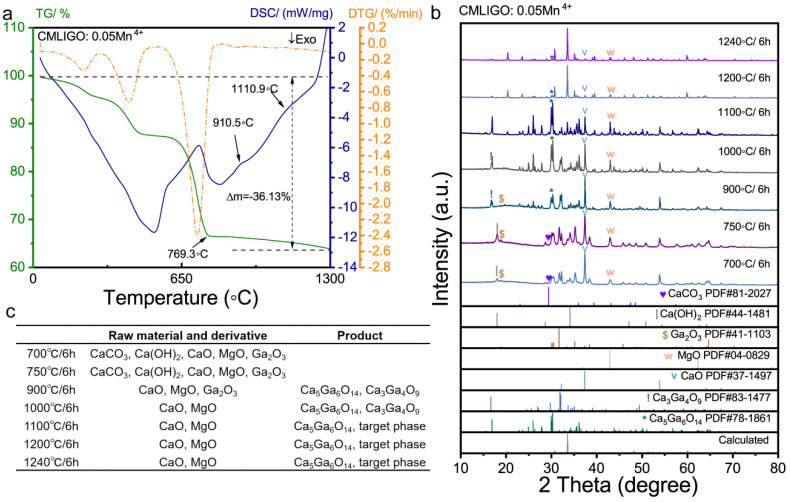
Structural evolution and phase composition of CMLIGO:0.05Mn^4+^ under different temperatures. (**a**) TG-DSC curves and DTG curve of the CMLIGO:0.05Mn^4+^. (**b**) XRD patterns of CMLIGO:0.05Mn^4+^ samples calcined at different temperatures, which are 700 °C, 750 °C, 900 °C, 1000 °C, 1200 °C, 1240 °C. (**c**) The phases identified in CMLIGO:0.05Mn^4+^ samples calcined at different temperatures, which are 700 °C, 750 °C, 900 °C, 1000 °C, 1200 °C, 1240 °C.

To investigate whether the addition of MgCl_2_, in addition to its fluxing effect, also influences the reaction pathway of the system—as it has been shown to do in Ca_14_Zn_6_Ga_10_O_35_ systems [52]—we performed thermal analysis on CMLIGO:0.05Mn^4+^. The results are presented in Figure 5a. The thermogravimetry (TG) curve in Figure 5a exhibits three loss steps, each of them corresponding to an endothermic peak in the differential scanning calorimetry (DSC) curve and a peak in the derivative thermogravimetric (DTG) curve.

The loss steps were assigned to specific reactions based on the decomposition temperatures of the raw materials, and the theoretical weight loss percentages for these reactions were calculated. Table 2 lists each loss step, its corresponding reaction, and both the theoretical and experimental weight loss percentages. It can be observed that the theoretical values are in good agreement with the experimental data. This indicates that the three distinct loss steps in the TG curve correspond sequentially to the following processes as temperature increases: (1) removal of crystalline water from the 4MgCO_3_·Mg(OH)_2_·5H_2_O; (2) decomposition of MgCO_3_, Mg(OH)_2_ and MnCO_3_ into respective oxides; (3) decomposition of CaCO_3_ and Li_2_CO_3_ into respective oxides.

The mass drift (Δm = −2.60%) observed in the TG curve at temperatures above 800 °C corresponds to a mass change of 0.27 mg, which falls within the range of normal baseline fluctuation for TG measurements.

The third loss step concluded at 769.3 °C, corresponding to the temperature at which all raw materials were converted to respective oxides. The DSC curve revealed two endothermic peaks above 769.3 °C, located in the temperature ranges of 876.9–952.7 °C and 1046.1–1191.9 °C, respectively. Figure 5b displays the XRD patterns of the uniformly mixed raw powder after calcination at different temperatures and the phases identified in each sample are listed in the table of Figure 5c. Both samples calcined at 700 °C and 750 °C for 6 h contain Ca(OH)_2_, which is attributed to the reaction of CaO with moisture from the air. It is noteworthy that the sample calcined at 750 °C contained a trace amount of CaCO_3_ (corresponding to the incomplete decomposition of CaCO_3_ observed in the TG curve) but did not contain any intermediate products. It was not until the sample was calcined at 900 °C that the intermediate products, Ca_5_Ga_6_O_14_ and Ca_3_Ga_4_O_9_, emerged without any CaCO_3_ remaining in the system. It demonstrates that CaCO_3_ cannot act as the calcium source for the formation directly. Instead, the reaction proceeds through CaO as the direct calcium source.

Ca_3_Ga_4_O_9_ [60,61] is similar to Ca_5_Ga_6_O_14_, consisting of alternating layers of GaO groups and CaO groups. Both Ca_3_Ga_4_O_9_ and Ca_5_Ga_6_O_14_ were identified in the sample annealed at 900 °C. However, the content of the Ca_3_Ga_4_O_9_ presented the following trend: m_900 °C/6 h_ > m_1000 °C/6 h_, and it disappeared in the sample calcined at 1100 °C. It indicates that the formation reactions of Ca_3_Ga_4_O_9_ and Ca_5_Ga_6_O_14_ occur between 750 °C and 900 °C. Subsequently, in the temperature range of 900 °C to 1000 °C, Ca_3_Ga_4_O_9_ decomposed and Ca_5_Ga_6_O_14_ was extensively synthesized. It should also be noted that the abundance of the Ca_5_Ga_6_O_14_, which was substantial in the sample treated at 1100 °C, decreased significantly in the sample treated at 1200 °C. Concurrently, the target phase, which was only present in minor amounts at 1100 °C, became the dominant phase in the sample annealed at 1200 °C for 6 h. It demonstrates that, within the temperature interval of 1100 °C to 1200 °C, the system consumes a large amount of the Ca_5_Ga_6_O_14_ to form the target phase CMGO. This transformation corresponds to the endothermic peak of the DSC curve between 1046.1 °C and 1191.9 °C.

In summary, with increasing temperature and in the absence of any flux, the CMLIGO:0.05Mn^4+^ system undergoes the following processes: (1) conversion of all raw materials into respective oxides; (2) formation of the layered intermediate phases Ca_5_Ga_6_O_14_ and Ca_3_Ga_4_O_9_; (3) decomposition of Ca_3_Ga_4_O_9_; and 4) eventual reaction of the remaining intermediate phases and residual oxide precursors to form the target cubic phase.

**Figure 6:**
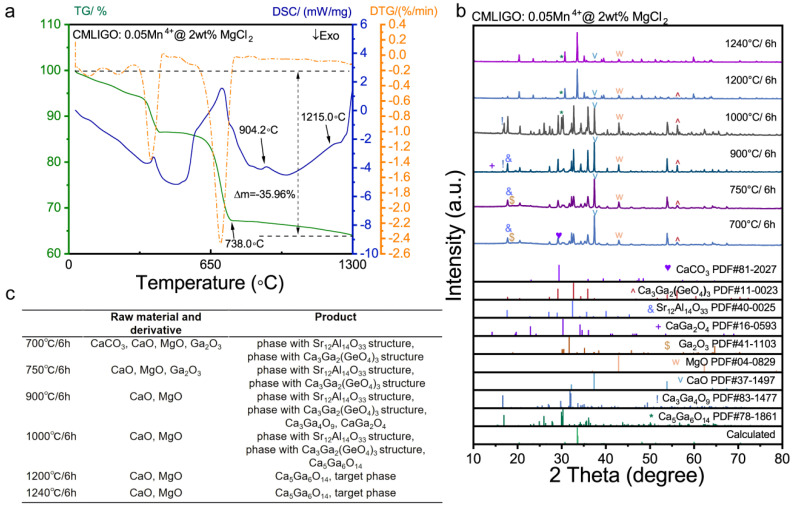
Structural evolution and phase composition of CMLIGO:0.05Mn^4+^@2 wt% MgCl_2_ under different temperatures. (**a**) TG-DSC curves and DTG curve of the CMLIGO:0.05Mn^4+^@2 wt% MgCl_2_. (**b**) XRD patterns of CMLIGO:0.05Mn^4+^@2 wt% MgCl_2_ samples calcined at different temperatures, which are 700 °C, 750 °C, 900 °C, 1000 °C, 1200 °C, 1240 °C. (**c**) The phases identified in CMLIGO:0.05Mn^4+^@2 wt% MgCl_2_ samples calcined at different temperatures, which are 700 °C, 750 °C, 900 °C, 1000 °C, 1200 °C, 1240 °C.

The TG-DSC curves for the CMLIGO:0.05Mn^4+^@2 wt% MgCl_2_ are shown in Figure 6a. Similar to CMLIGO:0.05Mn^4+^, the TG curve of CMLIGO:0.05Mn^4+^@2 wt% MgCl_2_ also exhibits three distinct weight loss steps. The theoretical weight loss percentages corresponding to the chemical reactions for each step were calculated. Table 3 lists the experimental weight loss percentages for each TG step, the chemical reactions occurring in that stage, and their corresponding theoretical weight loss percentages.

It is important to note that the addition of MgCl_2_ causes the raw material mixture to adsorb a significant amount of atmospheric moisture. The evaporation of adsorbed water, combined with the loss of crystalline water from 4MgCO_3_·Mg(OH)_2_·5H_2_O, contributes to the total weight loss observed in the first TG step. The theoretical weight loss from the dehydration of 4MgCO_3_·Mg(OH)_2_·5H_2_O alone is 3.59%. Therefore, the actual weight loss should be greater than this value, as confirmed by the experimental data. The second weight loss step corresponds to the decomposition of MgCO_3_, Mg(OH)_2_ and MnCO_3_, while the third weight loss step is attributed to the decomposition of CaCO_3_ and Li_2_CO_3_. The theoretical and experimental weight losses for these two steps are in excellent agreement. The TG curve of the CMLIGO:0.05Mn^4+^@2 wt% MgCl_2_ sample also shows a baseline drift within the margin of error (Δm = −3.16%) at temperatures above 800 °C.

It is noteworthy that the third loss step for CMLIGO:0.05Mn^4+^@2 wt% MgCl_2_ concluded at a lower temperature of 738.0 °C compared to the flux-free CMLIGO:0.05Mn^4+^. It indicates that the addition of MgCl_2_ facilitates the decomposition of CaCO_3_. Furthermore, two endothermic peaks were observed above 738.0 °C, within the ranges of 876.6–994.2 °C and 1113.6–1250.0 °C, respectively. The uniformly mixed precursor of CMLIGO:0.05Mn^4+^@2 wt% MgCl_2_ was calcined at different temperatures. The XRD patterns of these samples are presented in Figure 6b and the phases of intermediate products identified in each sample are listed in the table of Figure 6c.

CaGa_2_O_4_ [62], which appeared in small quantities in the sample heat-treated at 900 °C and disappeared after treatment at 1000 °C, belongs to the orthorhombic system. It is noticeable that intermediate products were already detected in the sample treated at 700 °C. Their powder diffraction data match well with Sr_12_Al_14_O_33_ (PDF#40-0025) and Ca_3_Ga_2_(GeO_4_)_3_ (PDF#11-0023), respectively. Although the raw mixture contains no Sr, Al, or Ge sources, it can be confirmed that reactions occur at 700 °C, forming intermediate products with structures isotypic to Sr_12_Al_14_O_33_ and Ca_3_Ga_2_(GeO_4_)_3_. Sr_12_Al_14_O_33_ [63] has a cubic structure. It comprises a three-dimensional network formed by AlO_4_ and SrO_6_ polyhedra. Ca_3_Ga_2_(GeO_4_)_3_ [64,65] is a common phosphor host material possessing a cubic garnet structure. The coexistence of CaCO_3_ and these intermediate products in the sample calcined at 700 °C implies that the addition of MgCl_2_ promotes the incorporation of Ca into the reaction system. This is further corroborated by the absence of CaCO_3_ in the sample treated at 750 °C, which aligns with the ending temperature (738.0 °C) of the third loss step in the TG curve of CMLIGO:0.05Mn^4+^@2 wt% MgCl_2_.

The intermediate product with the structure isotypic to Sr_12_Al_14_O_33_ formed as early as 700 °C and decomposed from 900–1000 °C. It exhibited a broad phase-formation temperature range. Similarly, the other intermediate product, isostructural to the Ca_3_Ga_2_(GeO_4_)_3_, was also observed at 700 °C and it remained stable up to 1200 °C. It means that both intermediate products possess wide stability ranges and can form at relatively low temperatures, i.e., the addition of MgCl_2_ provides a reaction pathway with a lower energy barrier. Besides that, the Ca_5_Ga_6_O_14_, which was already detectable at 900 °C in the flux-free CMLIGO:0.05Mn^4+^ system, was only observed in samples calcined at 1000 °C or higher temperatures after the introduction of MgCl_2_. This apparent delay in formation suggests that the addition of MgCl_2_ may suppress the formation of layered intermediate products.

**Figure 7:**
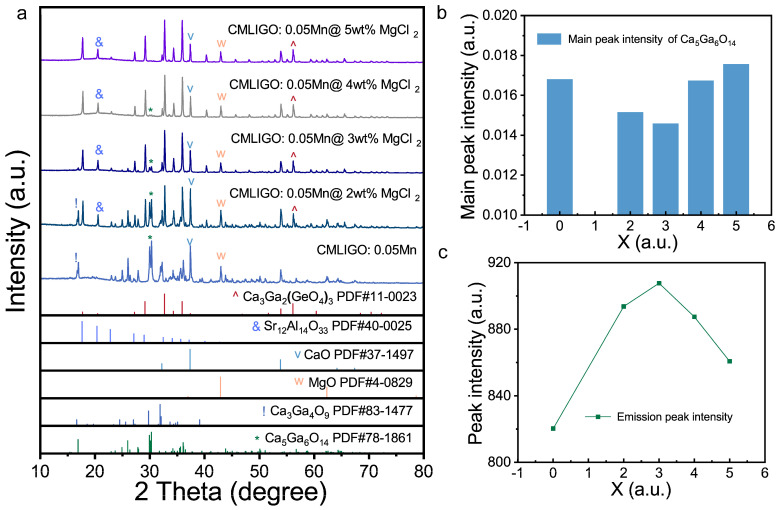
Influence of MgCl_2_ addition on the phase formation pathway, the ultimate phase composition and emission intensity. (**a**) XRD patterns of CMLIGO:0.05Mn^4+^@*x* wt% MgCl_2_ (*x* = 0, 2, 3, 4, 5) after calcination at 1000 °C for 6 h; (**b**) Intensity of the main XRD peak corresponding to Ca_5_Ga_6_O_14_ in the normalized patterns of the final calcined CMLIGO:0.05Mn^4+^@*x* wt% MgCl_2_ (*x* = 0, 2, 3, 4, 5), and (**c**) luminescence intensity of CMLIGO:0.05Mn^4+^@*x* wt% MgCl_2_ (*x* = 0, 2, 3, 4, 5) as a function of calcium carbonate addition.

To confirm the inhibitory effect of MgCl_2_ on the formation of layered intermediate products, CMLIGO:0.05Mn^4+^ samples with varying MgCl_2_ concentrations were calcined at 1000 °C. Their XRD patterns are presented in Figure 7a. Obviously, the content of the Ca_5_Ga_6_O_14_ in the samples treated at 1000 °C decreased with increasing amounts of MgCl_2_. There was no Ca_5_Ga_6_O_14_ detected in samples with a MgCl_2_ addition exceeding 4 wt%. Furthermore, Ca_3_Ga_4_O_9_, another layered intermediate product, was only present in the flux-free CMLIGO:0.05Mn^4+^ sample and CMLIGO:0.05Mn^4+^@2 wt% MgCl_2_ sample calcined at 1000 °C. The content of Ca_3_Ga_4_O_9_ was higher in the sample without MgCl_2_ than in the one with 2 wt% MgCl_2_ and it was absent when the addition of MgCl_2_ exceeded 2 wt%. These phenomena indicate that MgCl_2_ suppresses the formation of layered intermediate products.

Figure 7b displays the intensity of the main peak of Ca_5_Ga_6_O_14_ in normalized XRD patterns of the final calcined CMLIGO:0.05Mn^4+^@*x* wt% MgCl_2_ and the intensity as a function of *x* and Figure 7c shows the peak emission intensity of all samples as a function of *x*. The trends of the PL intensity and the Ca_5_Ga_6_O_14_ content are evidently opposite. Within the range of 0 to 3 wt% MgCl_2_, the PL intensity increases concomitantly with the decrease in the Ca_5_Ga_6_O_14_ content in the calcined samples. Since the Ca_5_Ga_6_O_14_ cannot be fully converted during the phase formation upon calcination, the content of it in the final samples reflects the total amount generated during the reaction. Thus, a reduction in the forming of Ca_5_Ga_6_O_14_ was accompanied by a corresponding increase in the photoluminescence intensity of the final sample. This indicates that the reduction in the content of layered intermediate phases (along with the increase in cubic intermediate products) within the system contributes to the enhancement of luminescence.

When the MgCl_2_ content increases further beyond 3 wt%, the Ca_5_Ga_6_O_14_ content exhibits a rebound, accompanied by a concurrent decrease in PL intensity. This phenomenon further indicates that the Ca_5_Ga_6_O_14_ content no longer shows a strict dependence on the MgCl_2_ concentration, reflecting that the inhibitory effect of MgCl_2_ becomes less effective above 3 wt%. It suggests that MgCl_2_ should be added within an appropriate concentration range.

Since the variation in the luminescence of the phosphors is able to reflect the change of the defect content within the crystal lattice [48,66], we propose the following hypothesis: when the MgCl_2_ addition is within the range of 0–3 wt%, the formation of layered intermediate products during the reaction will be suppressed, as evidenced by the reduced content of Ca_5_Ga_6_O_14_ in the final samples. At the same time, the defect content in the crystal lattice of the final samples will decrease, leading to an enhancement in the luminescence intensity. Further increasing the MgCl_2_ content, the efficacy in suppressing the layered phases will reduce, which is indicated by an increased Ca_5_Ga_6_O_14_ content after calcination and might be accompanied by a rise in the defect content of the final calcined samples. This hypothesis requires further substantiation through time-resolved photoluminescence spectroscopy.

### 3.4. Luminescence Properties

To investigate the effect of MgCl_2_ addition on the defect content in the samples, time-resolved photoluminescence (TRPL) spectra of the final calcined CMLIGO:0.05Mn^4+^ and CMLIGO:0.05Mn^4+^@3 wt% MgCl_2_ were measured and fitted using the following equation [67]:

(6) $$I\left(t\right)=A_{1}\exp\left(-\frac{t}{\tau_{1}}\right)+A_{2}\exp\left(-\frac{t}{\tau_{2}}\right)+y_{0}$$

**Figure 8:**
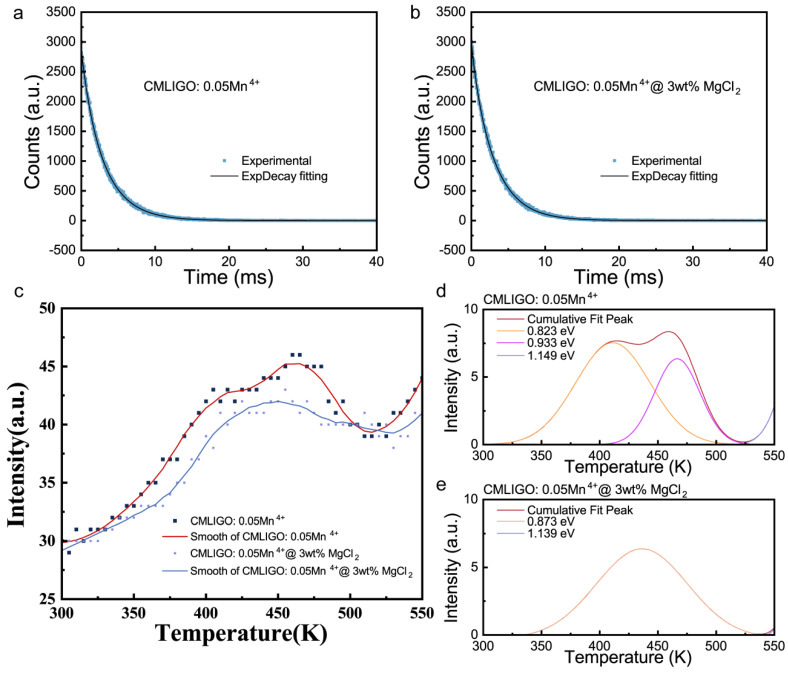
Effect of MgCl_2_ addition on non-radiative transitions and trap content in CMLIGO:0.05Mn^4+^. (**a**) Time-resolved photoluminescence spectra of CMLIGO:0.05Mn^4+^ and (**b**) CMLIGO:0.05Mn^4+^@3 wt% MgCl_2_. The solid lines represent the exponential fitting results of the time-resolved photoluminescence spectra. (**c**) Thermoluminescence (TL) glow curves of CMLIGO:0.05Mn^4+^ and CMLIGO:0.05Mn^4+^@3 wt% MgCl_2_. The scattered symbols represent the raw experimental data, and the solid lines represent the smoothed curves using a 15-point Savitzky–Golay filter. (**d**) TL glow curve fitting results for CMLIGO:0.05Mn^4+^ and (**e**) CMLIGO:0.05Mn^4+^@3 wt% MgCl_2_. Here, the light blue solid line corresponds to deep traps in the vicinity of 1.1 eV, as determined by fitting. The peaks of these deep traps lie above 550 K, whereas their contributions below 550 K are presented in the figure.

Here, τ_1_ represents the short decay component, which is attributed to non-radiative energy transfer arising from various quenching mechanisms; τ_2_ represents the long decay component, assigned to the ^2^E_2g_ → ^4^A_2g_ transition of Mn^4+^; A_1_ and A_2_ are pre-exponential functions, which are related to the concentration of species responsible for the transition or energy transfer in the material [68]. Figure 8a,b present the TRPL spectra and the corresponding fitting results. Table 4 summarizes the fitted data. Compared to CMLIGO:0.05Mn^4+^, the short decay component τ_1_ of CMLIGO:0.05Mn^4+^@3 wt% MgCl_2_ decreases, indicating a reduced distance between Mn^4+^ and quenching centers, which corresponds to the lattice contraction. Meanwhile, it can be observed that the pre-exponential function A_1_ of CMLIGO:0.05Mn^4+^@3 wt% MgCl_2_ decreases relative to that of CMLIGO:0.05Mn^4+^, while the pre-exponential function A_2_ increases compared to that of CMLIGO:0.05Mn^4+^. This suggests that, in contrast to CMLIGO:0.05Mn^4+^, the proportion of sites undergoing non-radiative transitions (quenching centers) is lower in CMLIGO:0.05Mn^4+^@3 wt% MgCl_2_ [69].

Figure 8c presents the thermoluminescence (TL) glow curves (scatter plots) and their corresponding smoothed results for CMLIGO:0.05Mn^4+^ and CMLIGO:0.05Mn^4+^@3 wt% MgCl_2_. The smoothed TL data were subsequently deconvoluted into Gaussian peaks, with the fitting results shown in Figure 8d,e for CMLIGO:0.05Mn^4+^ and CMLIGO:0.05Mn^4+^@3 wt% MgCl_2_, respectively. Based on the peak positions obtained from the deconvolution, the trap depths of the samples can be calculated using the Urbach equation [70]:

(7) $$E_{trap}=T_{m}/500$$

Here, E_trap_ represents the energy of trap depth and T_m_ denotes the peak temperature obtained from the TL glow curve fitting. The fitted peak positions and the corresponding calculated trap depths for CMLIGO:0.05Mn^4+^ and CMLIGO:0.05Mn^4+^@3 wt% MgCl_2_ are summarized in Table 5. Compared with CMLIGO:0.05Mn^4+^, the fitted peak at 0.8 eV (attributed to shallow traps) disappears in the modified CMLIGO:0.05Mn^4+^@3 wt% MgCl_2_, leaving only the fitted peaks associated with deep traps at approximately 0.9 eV and 1.1 eV [71,72]. This indicates that MgCl_2_ modification can alter the trap distribution in CMLIGO:0.05Mn^4+^, selectively passivating shallow-trap-related defects or reducing their concentration.

**Figure 9:**
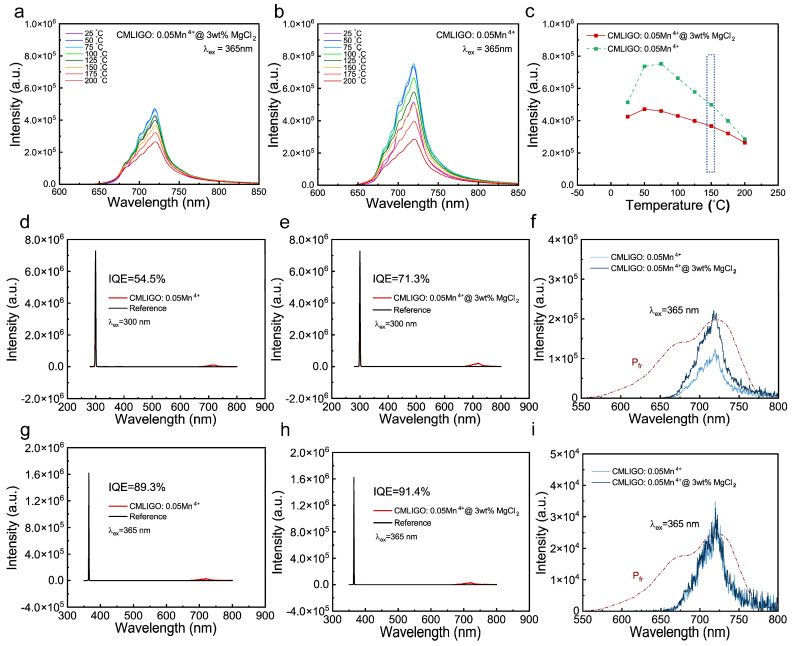
Effect of MgCl_2_ addition on temperature-dependent photoluminescence spectra and internal quantum efficiency. (**a**) Temperature-dependent photoluminescence spectra of CMLIGO:0.05Mn^4+^ and (**b**) CMLIGO:0.05Mn^4+^@3 wt% MgCl_2_. (**c**) Temperature-dependent peak emission intensity of CMLIGO:0.05Mn^4+^ and CMLIGO:0.05Mn^4+^@3 wt% MgCl_2_. The dashed box indicates the operating temperature range of pc-LEDs. (**d**) Quantum yield spectra of CMLIGO:0.05Mn^4+^ and (**e**) CMLIGO:0.05Mn^4+^@3 wt% MgCl_2_ under 300 nm excitation. (**f**) Emission bands of the quantum yield spectra for CMLIGO:0.05Mn^4+^ and CMLIGO:0.05Mn^4+^@3 wt% MgCl_2_ under 300 nm excitation, with the dash-dotted line representing the absorption spectrum of the photosensitive pigment P_fr_. (**g**) Quantum yield spectra of CMLIGO:0.05Mn^4+^ and (**h**) CMLIGO:0.05Mn^4+^@3 wt% MgCl_2_ under 365 nm excitation. (**i**) Emission bands of the quantum yield spectra for CMLIGO:0.05Mn^4+^ and CMLIGO:0.05Mn^4+^@3 wt% MgCl_2_ under 365 nm excitation, with the dash-dotted line representing the absorption spectrum of the photosensitive pigment P_fr_.

The temperature-dependent photoluminescence spectra of CMLIGO:0.05Mn^4+^ and CMLIGO:0.05Mn^4+^@3 wt% MgCl_2_, measured under excitation at the pc-LED operating wavelength of 365 nm, are shown in Figure 9a,b. Both samples exhibit anti-thermal quenching behavior in the temperature range of 25–50 °C, i.e., the emission intensity increases with increasing temperature. The integrated emission intensity as a function of temperature for both samples is presented in Figure 9c. At 150 °C (the operating temperature of pc-LEDs), the peak emission intensities of CMLIGO:0.05Mn^4+^ and CMLIGO:0.05Mn^4+^@3 wt% MgCl_2_ are 97.1% and 86.4% of their respective room-temperature values, indicating good thermal quenching resistance in both cases. Although the defect concentration is reduced in CMLIGO:0.05Mn^4+^ after MgCl_2_ modification, its thermal quenching resistance is inferior to that of the unmodified counterpart. This may suggest a correlation between the thermal quenching resistance (as well as the anti-thermal quenching behavior at low temperatures) and the shallow traps around 0.8 eV.

The quantum yields of CMLIGO:0.05Mn^4+^ and CMLIGO:0.05Mn^4+^@3 wt% MgCl_2_ were monitored at the peak excitation wavelength of 300 nm, and the results are shown in Figure 9d,e. The emission bands of the quantum yields are plotted together with the absorption spectrum of the photosensitive pigment P_fr_ in Figure 9f. The quantum yields of both samples were also measured under excitation at the pc-LED operating wavelength of 365 nm, and the results are presented in Figure 9g,h, with the corresponding emission bands and the absorption spectrum of Pfr shown in Figure 9i. The quantum yield curves include the excitation and emission spectra for both the tested samples and reference samples (BaSO_4_) in the integrating sphere. The internal quantum efficiency (IQE) can be calculated using the following formula [73]:

(8) $$\eta_{\mathrm{IQE}}=\frac{\int L_{S}}{\int E_{R}-\int E_{S}}$$

where L_S_ represents the emission spectrum of the test sample, while E_R_ and E_S_ denote the excitation spectra of the reference and test samples within the integrating sphere, respectively. The IQEs of CMLIGO:0.05Mn^4+^ and CMLIGO:0.05Mn^4+^@3 wt% MgCl_2_ under 300 nm excitation were calculated to be 54.5% and 71.3%, respectively. Under the same excitation wavelength (300 nm), the enhancement in PL intensity of CMLIGO:0.05Mn^4+^ induced by MgCl_2_ modification is also reflected in the IQE, which shows a significant increase upon modification. Under 365 nm excitation, the IQEs of CMLIGO:0.05Mn^4+^ and CMLIGO:0.05Mn^4+^@3 wt% MgCl_2_ were determined to be 89.3% and 91.4%, respectively, indicating that the positive effect of MgCl_2_ modification on the IQE of CMLIGO:0.05Mn^4+^ is still present.

As shown in Figure 9f,i, both the CMLIGO:0.05Mn^4+^ and CMLIGO:0.05Mn^4+^@3 wt% MgCl_2_ samples exhibit far-red emission in the range of 600–800 nm with a peak at 720 nm under both 300 nm and 365 nm excitation. These emission bands show a significant overlap and share the same peak position (720 nm) with the absorption spectrum of the photosensitive pigment P_fr_ [74,75]. The comparison with other far-red phosphors and their IQE values reported in the past decade is summarized in Table 6. These results demonstrate that the CMLIGO:0.05Mn^4+^@3 wt% MgCl_2_, with its suitable emission color and high IQE, meets the application requirements and is a promising far-red phosphor.

In summary, we found that MgCl_2_ addition within an appropriate range effectively suppresses the formation of two layered intermediate products (Ca_3_Ga_4_O_9_ and Ca_5_Ga_6_O_14_) in the CMLIGO:0.05Mn^4+^ system and promotes an alternative reaction pathway with a lower energy barrier: the precursors can react without fully decomposing into oxides and proceed through cubic intermediate products to form the final phase. Since the CMLIGO:0.05Mn^4+^ has a cubic crystal structure, the transformation from layered intermediate products (as in the absence of MgCl_2_) introduces more lattice defects. These defects act as quenching centers, degrading the luminescence. Conversely, the pathway via cubic intermediate products (facilitated by MgCl_2_) introduces far fewer defects. This results in a final product with lower defect concentration, higher crystal field strength, and a denser lattice, collectively leading to enhanced luminescent intensity. We can find a clear correlation between MgCl_2_-induced suppression of layered intermediate products and the improved luminescence. These phenomena demonstrate the effectiveness of steering the reaction pathway so that the precursors predominantly pass through intermediate products with a more similar structure, which can facilitate the formation of the final phase with a cubic structure and mitigate defect incorporation in the final samples and enhance its luminescent performance.

## 4. Conclusions

This work demonstrates that the addition of low-melting-point flux alters the reaction pathway of CMLIGO:0.05Mn^4+^ and significantly enhances its luminescence intensity. It suppresses the formation of intermediate products with a layered structure while promoting the conditions for cubic intermediate products with a closer structural resemblance to the target product.

This not only provides a reaction pathway with a lower energy barrier for the synthesis of CMLIGO:0.05Mn^4+^, facilitating the decomposition of the raw materials, but also reduces the defect concentration within the lattice of the final product, obtaining a final product with a compacter lattice. This effect is directly reflected in the enhancement of the luminescence intensity and quantum efficiency. The internal quantum efficiency (IQE) of CMLIGO:0.05Mn^4+^@3 wt% MgCl_2_ prepared using this method achieved a high internal quantum efficiency of 91.4%. The synthesized phosphor exhibits excellent thermal quenching resistance. It can be efficiently excited by 300 nm light, emitting far-red light with a peak at 720 nm, which matches the absorption spectrum of the plant photoreceptor P_fr_. These characteristics indicate its great potential for application in plant cultivation lighting. This work provides a new strategy for the synthesis and modification of composite oxide phosphors.

## Figures and Tables

**Table 1:** Calculated crystal field parameters and β_1_ of CMLIGO:0.05Mn^4+^ and CMLIGO:0.05Mn^4+^@*x* wt% MgCl_2_ (*x* = 0, 2, 3, 4, 5).

	Dq	Dq/B	B	C	β_1_
CMLIGO:0.05Mn^4+^	2114	2.87	736.7	2799.6	0.909
CMLIGO:0.05Mn^4+^@2 wt% MgCl_2_	2119	2.99	708.1	2859.3	0.902
CMLIGO:0.05Mn^4+^@3 wt% MgCl_2_	2117	3.00	705.0	2866.2	0.902
CMLIGO:0.05Mn^4+^@4 wt% MgCl_2_	2114	2.98	710.3	2854.7	0.903
CMLIGO:0.05Mn^4+^@5 wt% MgCl_2_	2119	2.99	708.1	2859.3	0.902

**Table 2:** TG loss steps, experimental weight loss percentages, corresponding reaction, and theoretical weight loss percentages for TG curve of CMLIGO:0.05Mn^4+^.

TG Loss Step	Experimental Weight Loss Percentage (wt%)	Corresponding Reaction	Theoretical Weight Loss Percentage (wt%)
loss step 1	4.43	The dehydration of 4MgCO_3_·Mg(OH)_2_·5H_2_O;	3.66
loss step 2	8.11	MnCO_3_ → MnO + CO_2_ MgCO_3_ → MgO + CO_2_ Mg(OH)_2_ → MgO + H_2_O	7.96
loss step 3	20.99	CaCO_3_ → CaO + CO_2_ Li_2_CO_3_ → Li_2_O + CO_2_	21.11

**Table 3:** TG loss steps, experimental weight loss percentages, corresponding reaction, and theoretical weight loss percentages for TG curve of CMLIGO:0.05Mn^4+^@2 wt% MgCl_2_.

TG Loss Step	Experimental Weight Loss Percentage (wt%)	Corresponding Reaction	Theoretical Weight Loss Percentage (wt%)
loss step 1	5.18	The dehydration of 4MgCO_3_·Mg(OH)_2_·5H_2_O; The evaporation of adsorbed water	≥3.59
loss step 2	8.07	MnCO_3_ → MnO + CO_2_ MgCO_3_ → MgO + CO_2_ Mg(OH)_2_ → MgO + H_2_O	7.80
loss step 3	19.37	CaCO_3_ → CaO + CO_2_ Li_2_CO_3_ → Li_2_O + CO_2_	20.70

**Table 4:** Fitting results of the fluorescence lifetimes for CMLIGO:0.05Mn^4+^ and CMLIGO:0.05Mn^4+^@3 wt% MgCl_2_.

	τ_1_ (ms)	τ_2_ (ms)	A_1_	A_2_	τ_avg_ (ms)
CMLIGO:0.05Mn^4+^	1.27	3.17	375.82	2473.45	3.06
CMLIGO:0.05Mn^4+^@3 wt% MgCl_2_	0.58	3.07	218.38	2783.23	3.03

**Table 5:** TL glow curve fitting results: peak positions and calculated trap depths for CMLIGO:0.05Mn^4+^ and CMLIGO:0.05Mn^4+^@3 wt% MgCl_2_.

	T_m_ (K)	E_trap_ (eV)
CMLIGO:0.05Mn^4+^	411.29	0.823
	466.58	0.933
	574.62	1.149
CMLIGO:0.05Mn^4+^@3 wt% MgCl_2_	436.31	0.873
	569.61	1.139

**Table 6:** Comparison of IQE values for recently reported phosphors.

Phosphor	IQE
CMLIGO:0.05Mn^4+^@3 wt% MgCl_2_	91.4% (this work)
Sr_9_Y_2_W_4_O_24_:0.005Mn^4+^	49.8% [76]
Li_2_MgTi_3_O_8_:0.01Cr^3+^, 0.1Zn^2+^	41.3% [23]
CaYMgSbO_6_:0.002Mn^4+^	51.5% [77]
Ca_2_GdTaO_6_:0.004Mn^4+^	33.0% [78]
Sr_2_LaSbO_6_:0.008Mn^4+^, 0.008Al^3+^	38.1% [79]
Sr_3_LiSbO_6_:0.003Mn^4+^	52.3% [80]
Ca_0.96_Zn_0.04_Al_12_O_19_:0.005Mn^4+^	67.1% [81]
SrMgAl_10_O_17_:0.003Cr^3+^	44.1% [24]
Ba_2_LaSbO_6_:0.0013Mn^4+^	20.2% [82]
Sr_2_GdSbO_6_:0.004Mn^4+^, 0.004 W^6+^	40.1% [83]
LiLaMgWO_6_:0.007Mn^4+^	69.1% [84]

## Data Availability

The original contributions presented in this study are included in the article/Supplementary Materials. Further inquiries can be directed to the corresponding author.

## Supplementary Materials

The following supporting information can be downloaded at: https://www.mdpi.com/article/10.3390/ma19071367/s1, Figure S1: (a–e) SEM images (magnified 1000 times) of CMLIGO:0.05Mn^4+^@*x* wt% MgCl_2_ with *x* = 0, 2, 3, 4, 5.
