# Supplementary figures and images for: A Novel Strategy for High Quantum Efficiency Composite Oxide Far-Red Phosphors: Ca14Mg5.94Li0.03In0.03Ga9.95O35:0.05Mn4+
Source: Materials (Basel). 2026 Mar 30;19(7):1367. doi: 10.3390/ma19071367 (PMC13075092; doi:10.3390/ma19071367)

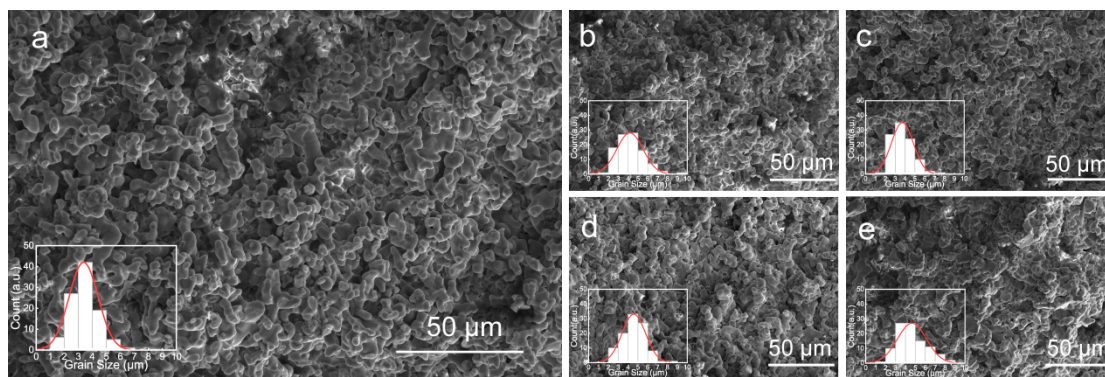

**Figure S1.** (a-e) SEM images(magnified 1000 times) of CMLIGO: 0.05Mn<sup>4+</sup>@x wt% MgCl<sub>2</sub> with x = 0, 2, 3, 4, 5).

Supplement: Supplementary file 1 [file materials-19-01367-s001.zip › materials-4191536-supplementary.pdf]
